# Effects of Adalimumab on Mitochondria of Psoriatic Mesenchymal Stem Cells

**DOI:** 10.1111/jcmm.70642

**Published:** 2025-06-06

**Authors:** Mariangela Di Vincenzo, Anna Campanati, Giulia Cannelonga, Federico Diotallevi, Giorgia Cerqueni, Andrea Marani, Saverio Marchi, Monia Orciani

**Affiliations:** ^1^ Histology‐Department of Clinical and Molecular Sciences Università Politecnica Delle Marche Ancona Italy; ^2^ Dermatologic Clinic‐Department of Clinical and Molecular Sciences Università Politecnica Delle Marche Ancona Italy; ^3^ Pharmacology‐Department of Biomedical Sciences and Public Health Università Politecnica Delle Marche Ancona Italy

**Keywords:** biological agents, inflammation, mesenchymal stem cells, mitochondria, oxidative stress, psoriasis

## Abstract

Psoriasis is a systemic immune‐mediated disorder involving multiple signalling pathways. Recent attempts to treat psoriasis involve monoclonal antibodies that block different inflammatory pathways. The monoclonal antibody Adalimumab (ADM) is one of the biologics that block the inflammatory cascade of TNF‐alpha. We previously demonstrated the involvement of mesenchymal stem cells (MSCs) in psoriasis pathogenesis and showed their responsiveness to ADM, highlighting their dual role as contributors and therapeutic targets. Mitochondrial dysfunction is increasingly recognised in psoriasis, contributing to oxidative stress, altered metabolism, and immune dysregulation. These mitochondrial changes drive chronic inflammation, keratinocyte hyperproliferation, and immune activation—hallmarks of psoriasis—making mitochondria a key focus for therapeutic strategies. In this study, we further investigated ADM's impact on mitochondrial morphology and function in MSCs. MSCs were isolated from the skin of psoriatic patients (PSO‐MSCs) and healthy controls (C‐MSCs), then exposed to H_2_O_2_ or LPS to mimic the oxidative and inflammatory environment of psoriasis. PSO‐MSCs were also treated with ADM for 72 h before mitochondrial analysis. Compared to C‐MSCs, PSO‐MSCs showed marked mitochondrial abnormalities. ADM treatment partially reversed these alterations, restoring mitochondrial parameters toward control levels under basal conditions. However, ADM failed to prevent mitochondrial dysfunction when additional stress (H_2_O_2_ or LPS) was introduced. In conclusion, ADM exerts a protective effect on mitochondrial health in MSCs from psoriatic patients, suggesting mitochondria are among its therapeutic targets. Nonetheless, ADM alone cannot counteract further environmental or inflammatory stressors, which may explain symptom relapse observed in clinical settings.

## 1 Introduction

Psoriasis is a chronic, systemic immune‐mediated disease and several players, including genetic, environmental, and immunological factors, have a role in its pathogenesis.

Regarding therapy, in addition to traditional systemic therapies, newer biologic agents that specifically target cytokines involved in the psoriasis inflammatory cascade are increasingly used. Tumour necrosis factor‐alpha (TNF‐α), in particular, plays a significant role in the development and persistence of psoriasis by promoting keratinocyte proliferation and stimulating T cell activation. To block these effects, the fully human monoclonal antibody Adalimumab (ADM) is employed to target TNF‐α [1].

Recently, mitochondrial function has gained more attention, based on three mechanisms that appear to correlate mitochondria and psoriasis: impaired lipid metabolism and oxidative stress, alterations in keratinocyte mitochondria after drug treatments, increase of mtDNA in the serum of psoriatic patients [2].

In chronically inflamed microenvironments, including psoriasis, pro‐inflammatory mediators can induce functional and morphological modifications of mitochondrial complexes, followed by irreversible mitochondrial remodelling, which in turn sustains inflammation in an increasing vicious circle [3].

In previous works, we have shown that mesenchymal stem cells (MSCs) are early involved in the onset and progression of psoriasis [4, 5, 6], and are responsive to drugs commonly used to treat psoriasis and which, until now, were thought to target only differentiated cells [7, 8, 9, 10].

These observations point out MSCs as new players in the regulation of the pathogenic mechanisms underlying psoriasis, as well as novel targets for therapeutic options.

No evidence is yet available on the effects of adalimumab on the mitochondria of MSCs; we showed that adalimumab influences the secretion of inflammatory cytokines by MSCs [10], but other cellular mechanisms have not been investigated.

Here, MSCs were isolated from the skin of psoriatic patients (PSO‐MSCs) and treated in vitro with adalimumab (ADM‐PSO‐MSCs); mitochondrial alterations, at both morphological (by live‐imaging and confocal microscopy analyses) and functional (by protein expression, ROS production, Ca^++^ uptake, apoptosis assays) levels, were then evaluated. MSCs were also isolated from the skin of control subjects (C‐MSCs) for comparative purposes. In addition, C‐ and PSO‐MSCs were treated with H_2_O_2_ or LPS to mimic the ROS‐stressed and inflamed microenvironment that characterises psoriasis.

## 2 Materials and Methods

### 2.1 Design of Study and Study Population

In this study, approved by the Università Politecnica delle Marche Ethical Committee (Protocol 2021 218) and conducted in accordance with the Declaration of Helsinki, a study group of six patients (three males, three females, mean age 47 ± 8.5) was enrolled together with a control group of six healthy subjects (three males, three females, mean age 57.2 ± 14.6), who were undergoing surgery for epidermal cysts. All participants gave their written consent prior to the study.

Patients were affected by stable, moderate to severe plaque psoriasis; the diagnosis was made by a trained dermatologist following the rule of 10: Body Surface Area (BSA) > 10%, Psoriasis Area Severity Index (PASI) > 10, Dermatology Life Quality Index (DLQI) > 10.

Disease severity was evaluated using the Psoriasis Area and Severity Index (PASI) score derived from the mean of two indices calculated by two independent trained dermatologists.

None of the patients reported symptoms or signs related to psoriatic arthritis. They were unresponsive to previous conventional treatments including topical applications of corticosteroids, retinoids, vitamin D3 derivatives, and systemic administration of cyclosporine, methotrexate, PUVA, UVA, UVB‐Nb, and they had not received systemic or topical therapies for almost three and 1 months, respectively.

### 2.2 Skin Samples Collection

Healthy subjects and psoriatic patients underwent skin punch biopsies (including lesional skin for psoriatic patients).

All punch biopsies were performed with a 5‐mm sterile cutaneous skin punch biopsy device (Gima, medical devices, s.r.l. Rome, Italy) after local anaesthesia with lidocaine 2%, and bioptic specimens were taken from the skin of the back for both psoriatic and healthy subjects.

### 2.3 Isolation and Characterisation of MSCs

C‐MSCs and PSO‐MSCs were isolated, cultured, and characterised as previously described [11, 12, 13] and according to the criteria by Dominici [14]. Briefly, after mincing, the samples were cultured with the mesenchymal stem cell growth medium bullet kit (Lonza Group Ltd). The morphology was evaluated by phase‐contrast microscopy (Leica DM IL; Leica Microsystems GmbH, Wetzlar, Germany). For all experiments, exponentially growing cells at the third passage and at a confluence of about 75% were used.

For immunophenotyping, 2.5 × 10^5^ cells were stained for 45 min with fluorescein isothiocyanate (FITC)‐conjugated antibodies (Becton Dickinson, Franklin Lakes, New Jersey, USA) against HLA‐DR, CD14, CD19, CD34, CD45, CD73, CD90 and CD105, and the data acquisition was carried out with CellQUEST software (Becton Dickinson).

For differentiative potential, cells were induced toward osteoblastic, chondrogenic, and adipogenic lineages using STEMPRO Osteogenesis, Chondrogenesis and Adipogenesis Kits (Life Technologies, Monza, Italy), respectively. Cells cultured in DMEM/F‐12 with 10% FBS were used as negative controls. Osteogenic, chondrogenic and adipogenic differentiations were, respectively, assessed by alkaline phosphatase (ALP) and von Kossa, Safranin O and Oil Red stainings.

### 2.4 Experimental Design

PSO‐MSCs were in vitro exposed to 10 μg/mL of ADM (Imraldi‐Adalimumab 40 mg) for 72 h before experiments (ADM‐PSO‐MSCs).

To induce stress or pro‐inflammatory conditions mimicking the psoriatic environment, cells (C‐MSCs and PSO‐MSCs) were treated with 200 μM of hydrogen peroxide (H_2_O_2_ Sigma‐Aldrich St. Louis, Missouri, USA) or with lipopolysaccharides (LPS—Sigma) for 1 or 2 h, respectively.

All the subsequent experiments were performed in triplicate on C‐MSCs, PSO‐MSCs and ADM‐PSO‐MSCs, before and after treatments with H_2_O_2_ and LPS (C‐MSCs‐H_2_O_2_, C‐MSCs‐LPS, PSO‐MSCs‐H_2_O_2_, PSO‐MSCs‐LPS, ADM‐PSO‐MSCs‐H_2_O_2_ and ADM‐PSO‐MSCs‐LPS).

### 2.5 Mitochondrial Morphological Analysis

Live images were acquired by a 3D Cell Explorer Nanolive microscope Fluo (Nanolive, Ecublens, Switzerland). Cells were transfected with mitochondria‐targeted cherry GFP (mito‐cherry) using PEI transfection reagent (Polysciences). Holo‐tomographic microscopy (HTM), in combination with epifluorescence, was performed on the 3D Cell‐Explorer Fluo. The acquisition was set for refractive index (RI) and epifluorescence (TRITC) every 2 min for a time‐lapse of 76 and 136 min for H_2_O_2_ and LPS treatment respectively. At the end of the experiment, image slices (−24 + 24) were exported using Steve software v.2.6 (Nanolive, Ecublens, Switzerland). Using Fiji‐ImageJ software (available online at https://imagej.net/Fiji), every RI and fluorescent frame was exported using z‐projection, maximum intensity algorithm, and the video constructed.

To evaluate the mitochondrial morphology, cells were incubated with 1:100 ATP5I primary antibody (ThermoFisher Scientific; Waltham, MA USA) for 2 h at RT followed by incubation with AlexaFluor488‐conjugated secondary antibody (ThermoFisher Scientific). Images were acquired with a Zeiss LSM510/Axiovert 200 M confocal microscope and then analysed by using Fiji‐ImageJ software. 2D analysis of mitochondrial morphology and network was performed by measuring form factor, aspect ratio, number of branches and branch junctions through the Fiji plugin ‘Mitochondria Analyser’ [15].

#### 2.5.1 Western Blot Analysis

The expression of proteins related to mitochondria morphology and apoptosis was quantified by Western Blot (WB) analysis as previously described [16]. Membranes were incubated with primary antibodies: dynamin‐related protein‐a DRP1 (Cell Signalling, Danvers, Massachusetts, USA, 1:1000), Bcl‐2 homologous antagonist/killer BAK1, apoptosis regulator BCL‐2 (Santa Cruz Biotechnology, Dallas, Texas, USA, 1:250), and GAPDH (Proteintech, Manchester, United Kingdom, 1:10000) and then with secondary antibodies goat anti‐rabbit HRP conjugated antibody (Invitrogen, Termofischer Scientific, 1:10000) or goat anti‐mouse HRP conjugated antibody (Bethyl Laboratories, Montgomery, TX, USA, 1:15000). Clarity Western ECL substrate (BioRad) was used for detection with Alliance Mini HD9 (Uvitec, Cambridge, UK). Densitometric analysis was performed by using Fiji‐ImageJ software.

### 2.6 Superoxide Detection by MitoSOX

The production of superoxide by mitochondria was measured by MitoSOX Red (Invitrogen, Termofisher Scientific) following the manufacturer's instructions. Fluorescence intensities were detected with a FACSCalibur Flow Cytometer: 3‐Colour (Becton Dickinson, East Rutherford, New Jersey, USA). Cells were gated according to physical parameters and histograms developed by using FlowJo Software (available online at https://www.flowjo.com/) with the percentage of positive events.

### 2.7 Mitochondrial Ca^2+^ Uptake Measurement

The mitochondrial calcium uptake was determined using jellyfish photoprotein aequorin‐based probes, gently gifted by Prof. P. Pinton (Signal Transduction's lab), as previously described [17, 18]. The light signal, detected with a photon counting detector (Hamamatsu Photonics, Japan), was collected and converted into [Ca^2+^] values by an algorithm based on the Ca^2+^ response curve of aequorin at physiological conditions.

### 2.8 Cell Viability Assay/Apoptosis Assay

5 × 10^5^cells were stained with 1 μM SYTOX AADvanced Dead Cell Stain (Thermofisher Scientific) for 15 min. Data were acquired with a FACSCalibur Flow Cytometer. Cells were gated according to physical parameters, and histograms were developed by using FlowJo Software with the percentage of positive events.

### 2.9 Statistical Analysis

GraphPad Prism 6 Software was used for statistical analysis. Data are expressed as the mean ± standard deviation (SD). For parametric analysis, all experimental groups were first tested for normal distribution by the Shapiro–Wilk test [19] and comparison between 2 groups was performed by unpaired Student's t‐test. For multiple comparisons, the two‐way ANOVA test (Tukey's multiple comparisons test) was used. Significance was set at *p* value < 0.05.

## 3 Results

### 3.1 Isolation and Characterisation of MSCs

**FIGURE 1:**
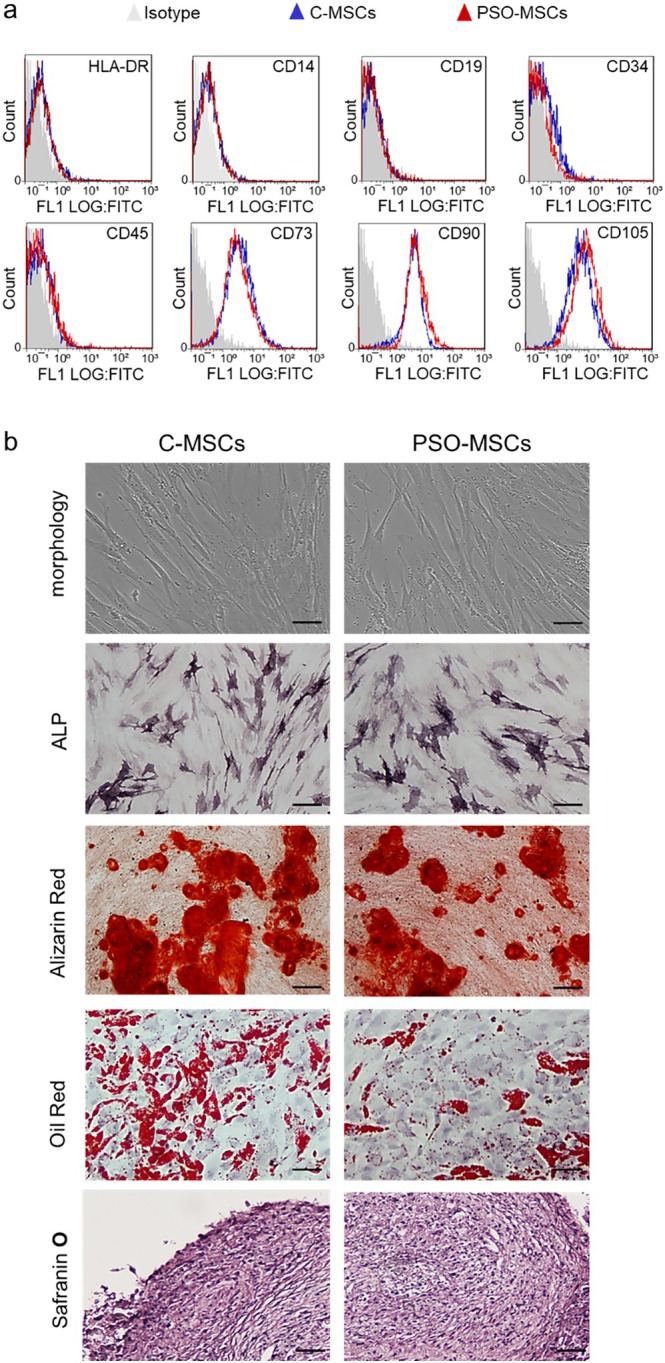
Mesenchymal stem cells (MSCs) characterization. (a) Representative Flow Cytometer analyses of MSCs derived from control skin biopsies (C‐MSCs) and psoriatic skin biopsies (PSO‐MSCs) surface antigen expression. Solid grey histograms refer to the negative control (IgG1 isotype control‐FITC labelled). (b) Phase‐contrast images of C‐MSCs and PSO‐MSCs after 21 days of culture (scale bar, 100 μm); representative images of osteogenic differentiation assessment by alkaline phosphatase (ALP) reaction (scale bar 100 μm) and Alizarin Red staining (scale bar 100 μm) after 7 and 21 days of differentiation respectively; adipocyte differentiation by Oil Red staining (scale bar 100 μm) after 21 days and chondrogenic differentiation by Safranin‐O coloration (scale bar 100 μm) after 21 days.

Cells, isolated from control and psoriatic skin, fully met the criteria identified by Dominici [14] for the definition of MSCs, such as plastic adherence, stem‐like immunophenotype, and differentiation capability toward osteogenic, adipogenic and chondrogenic lineages (Figure 1).

### 3.2 Adalimumab Affects Mitochondrial Morphology and Function

**FIGURE 2:**
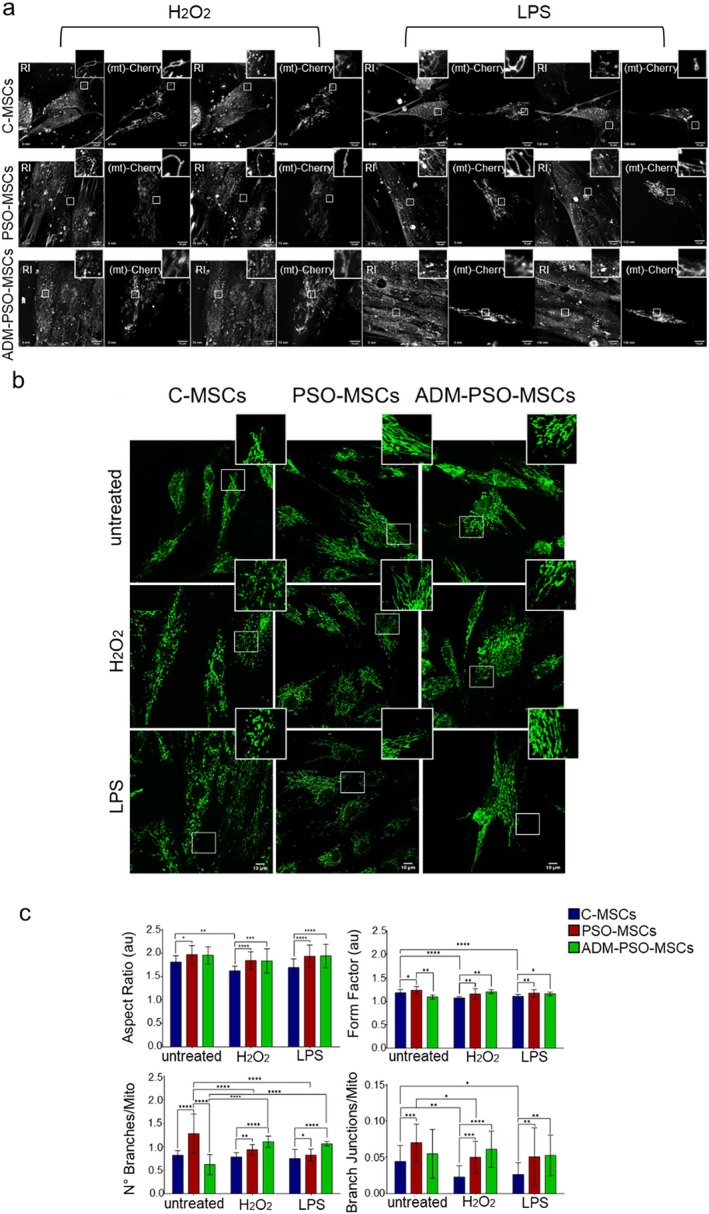
Alterations of mitochondrial morphology in PSO‐MSCs and effects of ADM treatment. (a) Live cell imaging 3D holotomographic (RI) and epifluorescent (mito‐cherry‐positive) imaging of C‐MSCs, PSO‐MSCs and ADM‐PSO‐MSCs mitochondrial structure (60X magnification). In the upper inset, enlargement of a mitochondrion portion at the start and the end of the treatments with H_2_O_2_ and LPS (b) Representative confocal images of the mitochondrial structure of C‐MSCs, PSO‐MSCs and ADM‐PSO‐MSCs before (untreated) and after H_2_O_2_ and LPS treatments (64X magnification). In the upper inset, an enlargement of a mitochondrion portion. (c) Analysis of mitochondria morphology (Aspect Ratio and Form Factor) and mitochondrial network (Branches and Branch Junctions) of C‐MSCs, PSO‐MSCs and ADM‐PSO‐MSCs before and after treatments (H_2_O_2_, LPS). Two‐way ANOVA; *p < 0.05, **p < 0.01, ***p < 0.001, ****p < 0.0001.

**FIGURE 3:**
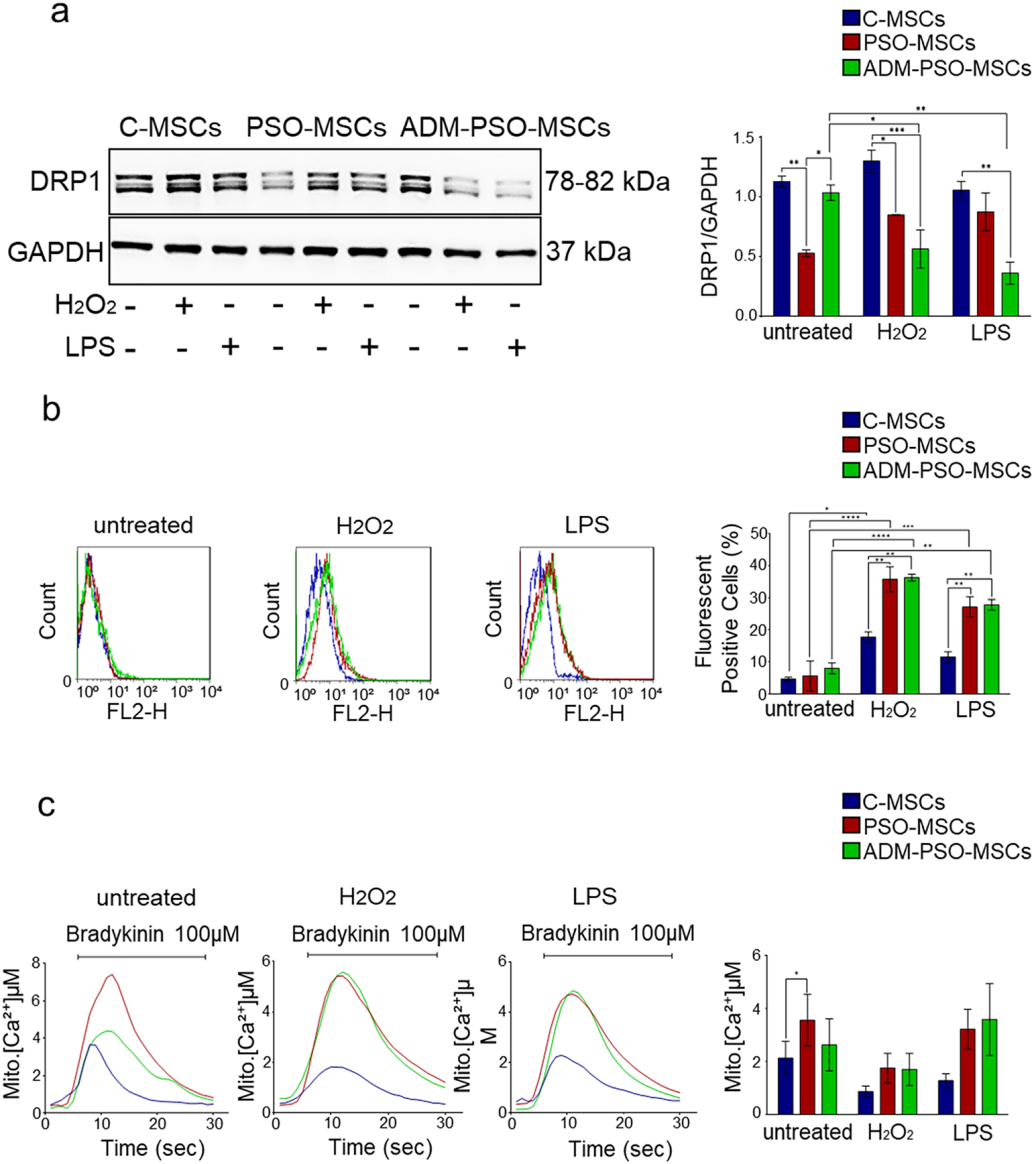
Dysregulated DRP1 levels, mitochondrial ROS production, and Ca^2+^ uptake in ADM‐PSO‐MSCs. (a) Representative images of DRP1 protein expression of C‐MSCs, PSO‐MSCs and ADM‐PSO‐MSCs before (untreated) and after treatments with H_2_O_2_ and LPS. The graph represents the densiometric analysis referred to GAPDH of DRP1. Two‐way ANOVA; *p < 0.05, **p < 0.01, ***p < 0.001 (b) Representative Flow Cytometer fluorescence graphs of MITOSOX of C‐MSCs (blue), PSO‐MSCs (red) and ADM‐PSO‐MSCs (green) before and after treatments with H_2_O_2_ and LPS. The graph represents the analysis of fluorescent positive cells percentage. Two‐way ANOVA; *p < 0.05, **p < 0,01, ***p < 0,001, ****p < 0,0001. (c) Mitochondrial Ca^2+^ homeostasis in C‐MSCs (blue), PSO‐MSCs (red) and ADM‐PSO‐MSCs (green) before (untreated) and after H_2_O_2_ and LPS treatments. The graph shows the statistical analysis of [Ca^2+^] expressed in μM. Two‐way ANOVA; *p < 0.05, **p < 0,01, ***p < 0,001, ****p < 0,0001.

As displayed in the selected frames of Figures 2a and 3d holotomographic microscopy in combination with epifluorescence showed that PSO‐MSCs were characterised by more filamentous and interconnected mitochondria than C‐MSCs at baseline (Minute 0). Treatment with ADM restored the mitochondrial structure of PSO‐MSCs, which appears similar to that of the control group. However, after H_2_O_2_ and LPS stimuli, ADM‐PSO‐MSCs did not undergo extensive mitochondrial fragmentation like C‐MSCs and showed mitochondrial structures resembling those of untreated psoriatic cells, which exhibited great resistance to H_2_O_2_ and LPS stimuli, maintaining a more interconnected mitochondrial profile (Figure 2a).

To confirm these observations and quantify mitochondrial variations, fluorescence images of the mitochondrial structure were acquired using a confocal microscopy‐based approach, as shown in Figure 2b. The analysis of the mitochondria morphology (Form Factor and Aspect Ratio) and mitochondrial interconnectivity (Branches number and Branch Junctions) confirmed that treatment with adalimumab led to a reduction in all these parameters, indicating that AD‐PSO‐MSCs showed less filamentous and less interconnected mitochondria than PSO‐MSCs, restoring a morphology more similar to that observed in C‐MSCs.

However, after H_2_O_2_‐ and LPS‐induced stress, mitochondria of ADM‐PSO‐MSCs showed an increase for all the selected parameters (Form Factor, Aspect Ratio, Branches number and Branch Junctions) that reached levels not significantly different from those observed in PSO‐MSCs.

To understand the molecular link between the different mitochondrial morphology and behaviour observed in C‐ and PSO‐MSCs, the expression of fission‐ and fusion‐related proteins was tested. Among the proteins analysed, only DRP1 was differently expressed by C‐ and PSO‐MSCs under resting conditions and was thus further considered to evaluate the effects of adalimumab and stressful insults. ADM treatment was able to increase its expression at baseline but, under stress conditions, the level of expression of DRP1 plummeted regardless of ADM treatment (Figure 3a).

Notably, the same trend (restoration of conditions similar to the physiological ones measured in C‐MSCs before the insult with stressful stimuli) was observed during the analysis of other mitochondrial functions. ADM was unable to counteract ROS production in response to both H_2_O_2_ and LPS (Figure 3b). The amount of mitochondrial ROS production was significantly higher in PSO‐MSCs than in C‐MSCs, regardless of ADM treatment.

As indicated in Figure 3c, mitochondria from PSO‐MSCs displayed higher [Ca^2+^] than C‐MSCs, both under resting condition and after treatment with H_2_O_2_ and LPS, indicating a superior ability of psoriatic cells to accumulate Ca^2+^ within their mitochondrial matrix. Treatment with ADM restored normal mitochondrial Ca^2+^ uptake only under resting conditions.

**FIGURE 4:**
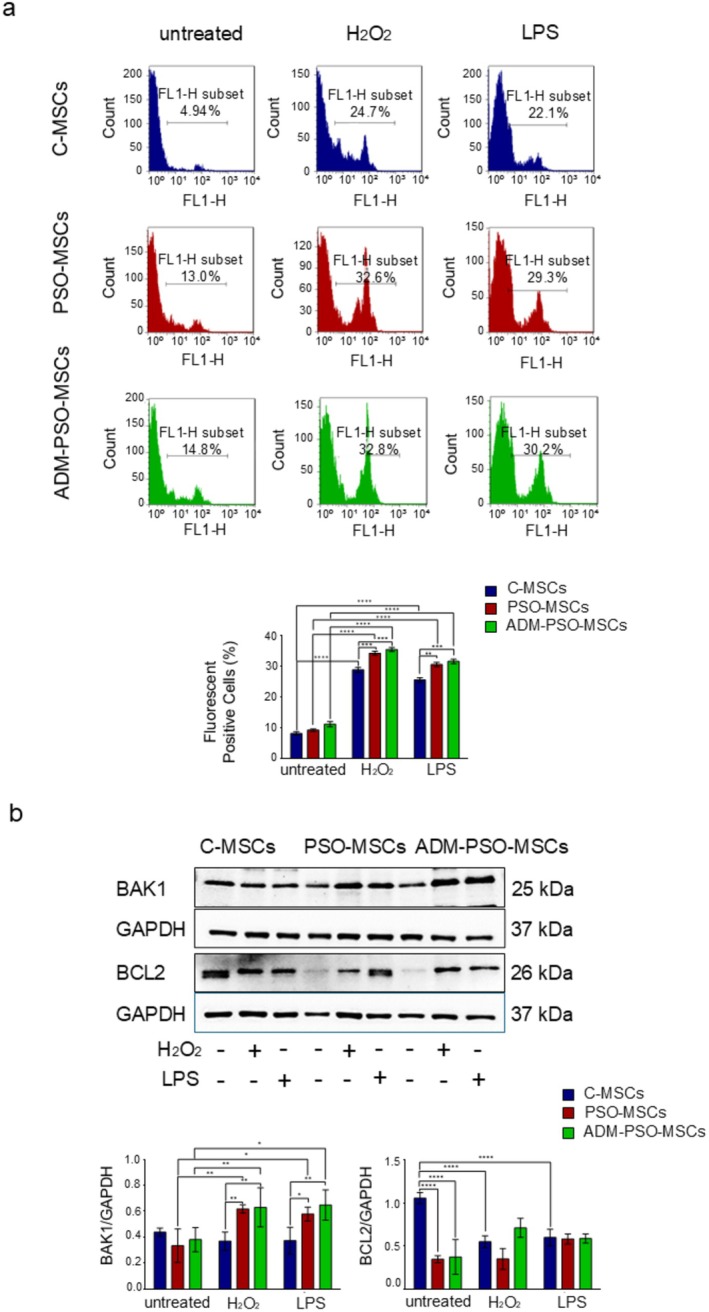
Effects of ADM on PSO‐MSCs cell death. (a) Representative Flow Cytometer fluorescence graphs of C‐ MSCs (blue), PSO‐MSCs (red) and ADM‐PSO‐MSCs (green), before (untreated) and after treatments (H_2_O_2_; LPS). The graph represents the analysis of SYTOX positive cells percentage. Two‐way ANOVA; **p < 0,01,***p < 0,001****p < 0,0001. (b) Representative images of BAK1, BCL2 and GAPDH expression of C‐MSCs, PSO‐MSCs and ADM‐PSO‐MSCs before and after treatments with H_2_O_2_ and LPS. The graphs below represent the densiometric analysis referred to GAPDH of each protein. Two‐way ANOVA; *p < 0.05, **p < 0,01, ***p < 0,001, ****p < 0,0001.

Flow cytometric analysis of cell viability, reported in Figure 4a, showed that the percentage of the apoptotic ADM‐PSO‐MSCs after H_2_O_2_ and LPS treatments was not significantly different from that observed in PSO‐MSCs and still higher than C‐MSCs. These data were straightened by WB analysis of pro‐apoptotic and anti‐apoptotic proteins related to the mitochondrial apoptotic pathway.

ADM exerted no effects: the expression level of these proteins by PSO‐MSCs did not change after pharmacological treatment.

Notably, BAK1 expression was almost the same in C‐ and PSO‐MSCs, while BCL2 was downregulated in PSO‐MSCs under resting conditions. Stimulation with H_2_O_2_ or LPS massively activated the pro‐apoptotic regulator (BAK1) and further downregulated the anti‐apoptotic factor (BCL2) in psoriatic cells (Figure 4b).

## 4 Discussion

Adalimumab (ADM) is a fully human monoclonal antibody against TNF‐α, used in the treatment of moderate to severe psoriasis. By binding to TNF‐α, ADM prevents its interaction with cell surface TNF receptors, thereby inhibiting a range of downstream biological responses, including inflammation, cell proliferation, and apoptosis [20, 21].

Inflammation and apoptosis are key pathogenic mechanisms in psoriasis and are closely linked to mitochondrial function. Recent studies have suggested a role for mitochondria in the onset of psoriasis [22], and it has been shown that several anti‐psoriatic treatments, such as phototherapy, can improve mitochondrial quality control and restore cellular redox status [2, 23].

Despite this growing evidence, no studies to date have explored the specific effects of ADM on the mitochondria of MSCs, which are involved early in psoriasis pathogenesis and have shown responsiveness to currently used therapies, positioning them as potential novel therapeutic targets.

This study aimed to evaluate the effects of ADM on the mitochondria of MSCs isolated from the skin of psoriatic patients (PSO‐MSCs), with MSCs derived from healthy skin (C‐MSCs) used as controls.

Prior to ADM treatment, PSO‐MSCs exhibited more elongated and interconnected mitochondria compared to C‐MSCs. Moreover, exposure to H_2_O_2_ or LPS‐induced stress did not alter mitochondrial morphology in PSO‐MSCs, suggesting that these mitochondria may have developed resistance to stress, possibly as an adaptive response to the chronic inflammatory environment characterised of psoriasis.

ADM treatment restored normal mitochondrial morphology in PSO‐MSCs under resting conditions. However, under stress conditions, ADM did not prevent mitochondrial alterations; in fact, additional stress reestablished the pathological levels of mitochondrial form factor, aspect ratio, number of branches, and branch junctions.

Since mitochondrial function is closely linked to its structural organisation, the expression of proteins involved in mitochondrial fusion and fission was analysed. The results showed a downregulation of DRP1 in PSO‐MSCs, consistent with previous studies reporting reduced DRP1 expression in psoriatic keratinocytes [24]. At baseline, treatment with ADM increased DRP1 expression to physiological levels; however, this effect was completely abolished following stimulation with H_2_O_2_ and LPS.

Reduced DRP1 expression is typically associated with the low apoptotic rate observed in keratinocytes, a trend not mirrored in PSO‐MSCs. This apparent contradiction may be explained by the more complex regulation of DRP1 in undifferentiated cells. Pernaute et al. [25] reported that mouse stem cells have a lower mitochondrial apoptotic threshold, making them more sensitive to cell death. Specifically, they demonstrated that this heightened apoptotic susceptibility was linked to a decrease in mitochondrial fission due to reduced DRP1 activity, a finding consistent with our observations.

Notably, ADM did not prevent apoptosis in PSO‐MSCs. This result partially contrasts with that of Grabarek [26], who showed that ADM exposure in keratinocytes significantly increased the activity of caspase‐3 and caspase‐9 compared to untreated controls. Caspase‐3, in turn, activates a cascade of caspases responsible for the proteolytic cleavage of cellular enzymes and structural proteins—a hallmark of the intrinsic mitochondrial apoptotic pathway. Therefore, while ADM appears to induce apoptosis via the mitochondrial pathway in keratinocytes, this effect is not replicated in undifferentiated cells such as MSCs.

Although BAK and BCL2 expression levels differed between C‐MSCs and PSO‐MSCs—again pointing to the involvement of the mitochondrial apoptotic pathway—ADM treatment did not produce any significant changes in their expression before or after administration.

We previously demonstrated that ADM enhances superoxide dismutase (SOD) activity, thereby reducing oxidative damage in MSCs. However, other antioxidant systems, such as glutathione‐S‐transferase and catalase, responded to ADM with a general reduction in activity. This led to an overall decrease in total oxyradical scavenging capacity, particularly against hydroxyl radicals and peroxynitrite [10].

Our recent findings strengthen the evidence that the intense pro‐oxidant pressure experienced by psoriatic MSCs can be only partially mitigated by ADM administration.

Increased calcium uptake is recognised as a key event in keratinocyte differentiation [27, 28]. Elevated intracellular calcium levels were also observed in PSO‐MSCs; however, treatment with ADM reversed this effect only under baseline conditions, not following H_2_O_2_‐ or LPS‐induced stress. Currently, there are no published data on the potential effects of ADM on calcium uptake in psoriasis, making it difficult to assess the divergent mechanisms between differentiated cells and MSCs.

In conclusion, this study further supports the critical role of MSCs in the early pathogenesis of psoriasis, highlighting their potential as targets for innovative therapeutic strategies. Within this framework, evaluating drug efficacy on MSCs becomes a key consideration. Our data suggest that ADM exerts effects on MSCs, but these effects are insufficient to fully restore normal cellular function or resilience to stress. This conclusion aligns with clinical observations showing that while ADM treatment leads to symptomatic improvement in patients, psoriasis may relapse under acute stress conditions.

Moreover, our findings point to mitochondrial dysfunction as a contributing factor in psoriasis pathogenesis, offering preliminary insights into aberrant pathways and signalling molecules that may drive disease progression.

## Author Contributions

**Mariangela Di Vincenzo:** formal analysis (lead), investigation (lead), methodology (lead), writing – original draft (equal). **Anna Campanati:** conceptualization (equal), validation (equal), writing – original draft (equal), writing – review and editing (lead). **Giulia Cannelonga:** formal analysis (lead), investigation (equal), methodology (equal). **Federico Diotallevi:** formal analysis (equal), investigation (equal). **Giorgia Cerqueni:** formal analysis (equal), investigation (equal). **Andrea Marani:** methodology (equal). **Saverio Marchi:** conceptualization (lead), funding acquisition (lead), supervision (lead), writing – original draft (lead), writing – review and editing (equal). **Monia Orciani:** conceptualization (lead), funding acquisition (lead), project administration (lead), supervision (lead), writing – original draft (lead), writing – review and editing (lead).

## Ethics Statement

This study was performed in line with the principles of the Declaration of Helsinki. Approval was granted by the Ethics Committee of Università Politecnica delle Marche (23‐09‐2021; No 2021‐218).

## Consent

Informed consent was obtained from all individual participants included in the study.

## Conflicts of Interest

The authors declare no conflicts of interest.

## Supporting information


**Video S1.** Live cell imaging 3D holotomographic (RI) of C‐MSCs (60X magnification). Treated with H_2_O_2_ for 1 h.

**Video S2.** Live cell imaging epifluorescent (mito‐cherry‐positive) imaging of C‐MSCs mitochondrial structure (60X magnification) treated with H_2_O_2_ for 1 h.

**Video S3.** Live cell imaging 3D holotomographic (RI) of C‐MSCs (60X magnification). Treated with LPS for 2 h.

**Video S4.** Live cell imaging epifluorescent (mito‐cherry‐positive) imaging of C‐MSCs mitochondrial structure (60X magnification) treated with LPS for 2 h.

**Video S5.** Live cell imaging 3D holotomographic (RI) of PSO‐MSCs (60X magnification). Treated with H_2_O_2_ for 1 h.

**Video S6.** Live cell imaging epifluorescent (mito‐cherry‐positive) imaging of PSO‐MSCs mitochondrial structure (60X magnification) treated with H_2_O_2_ for 1 h.

**Video S7.** Live cell imaging 3D holotomographic (RI) of PSO‐MSCs (60X magnification). Treated with LPS for 2 h.

**Video S8.** Live cell imaging epifluorescent (mito‐cherry‐positive) imaging of PSO‐MSCs mitochondrial structure (60X magnification) treated with LPS for 2 h.

**Video S9.** Live cell imaging 3D holotomographic (RI) of ADM‐PSO‐MSCs (60X magnification). Treated with H_2_O_2_ for 1 h.

**Video S10.** Live cell imaging epifluorescent (mito‐cherry‐positive) imaging of ADM‐PSO‐MSCs mitochondrial structure (60X magnification) treated with H_2_O_2_ for 1 h.

**Video S11.** Live cell imaging 3D holotomographic (RI) of ADM‐PSO‐MSCs (60X magnification). Treated with LPS for 2 h.

**Video S12.** Live cell imaging epifluorescent (mito‐cherry‐positive) imaging of ADM‐PSO‐MSCs mitochondrial structure (60X magnification) treated with LPS for 2 h.

## Data Availability

All data generated or analysed during this study are included in this published article (Video [Link], [Link], [Link], [Link], [Link], [Link], [Link], [Link], [Link], [Link], [Link], [Link]).
